# A 3 dimensional assessment of the depth of tumor invasion in microinvasive tongue squamous cell carcinoma - A case series analysis

**DOI:** 10.4317/medoral.20391

**Published:** 2015-10-09

**Authors:** Aditi Amit-Byatnal, Jayalakshmi Natarajan, Satish Shenoy, Asha Kamath, Keith Hunter, Raghu Radhakrishnan

**Affiliations:** 1 Post Graduate Residents, Department of Oral Pathology and Microbiology, Manipal College of Dental Sciences, Manipal, Manipal University, Karnataka, India; 2 Professor, Department of Aeronautical and Automobile Engineering, Manipal Institute of Technology, Manipal, Karnataka, India; 3 Assistant Professor, Department of Community Medicine, Kasturba Medical College, Manipal, Manipal University, Karnataka, India; 4 Clinical Senior Lecturer, Department of Oral and Maxillofacial Pathology, School of Clinical Dentistry, University of Sheffield, UK; 5 M.D.S., PhD Professor, Oral Pathology, Manipal University, India and Marie Curie International Incoming Fellow, Department of Oral and Maxillofacial Pathology, University of Sheffield, UK

## Abstract

**Background:**

Accurate assessment of the depth of tumor invasion (DI) in microinvasive squamous cell carcinoma (MISCC) of the tongue is critical to prognosis. An arithmetic model is generated to determine a reliable method of measurement of DI and correlate this with the local recurrence.

**Material and Methods:**

Tumor thickness (TT) and DI were measured in tissue sections of 14 cases of MISCC of the tongue, by manual ocular micrometer and digital image analysis at four reference points (A, B, C, and D). The comparison of TT and DI with relevant clinicopathologic parameters was assessed using Mann Whitney U test. Reliability of these methods and the values obtained were compared and correlated with the recurrence of tumors by Wilcoxon Signed Ranks Test. 3D reconstruction of the lesion was done on a Cartesian coordinate system. X face was on the YZ plane and Z face was on the XY plane of the coordinate system.

**Results:**

Computer generated 3D model of oral mucosa in four cases that recurred showed increased DI in the Z coordinate compared to the XY coordinate. The median DI measurements between XY and Z coordinates in these cases showed no significant difference (Wilcoxon Signed Ranks Test, *p* = 0.068).

**Conclusions:**

The assessment of DI in 3 dimensions is critical for accurate assessment of MISCC and precise DI allows complete removal of tumor.

**Key words:**Depth of invasion, tumor thickness, microinvasive squamous cell carcinoma, tongue squamous cell carcinoma.

## Introduction

Tongue squamous cell carcinoma (TSCC) is a common intraoral malignancy accounting for 25-40% of oral squamous cell carcinoma (OSCC) (1). While TSCCs diagnosed early have favorable prognosis, survival rates decline steadily with increasing age and advanced disease stage. Local recurrence of the tumor is one of the more common causes of treatment failure in patients with TSCC (1). Many parameters are taken into consideration to predict the recurrence and survival rate, including age, gender, habits, resection margins, tumor staging, histologic grading, depth of tumor invasion, occult nodal metastasis, perineural and lymphovascular invasion. Determination of the depth of tumor invasion (DI) is critical in micro invasive squamous cell carcinoma (MISCC) of the tongue due to the presence of excessive vascularity and increased propensity for regional lymph node metastasis.

MISCC is a cancer that infiltrates the superficial compartment of the lamina propria (2) and is defined as an invasive squamous cell carcinoma that extends into the stroma by < 0.5 mm, from the adjacent non-neoplastic epithelial basement membrane. The diagnosis of micro invasion is thus primarily histopathologic (3). Two of the most important characteristics of any epithelial malignancy that determine its local invasion are the thickness of tumor (TT) and the depth of invasion (DI) (4). Besides helping the clinician to plan a conservative surgical treatment protocol, microscopic determination of DI is considered to be crucial as it may have prognostic implication. This study was carried out utilizing two of the commonly available methods to measure the TT and DI in MISCC. From this a computer assisted 3-dimensional (3D) model of the oral mucosal reconstruct was generated to measure the DI in MISCC. This approach was tested in a series of cases of MISCC of tongue to correlate the findings with local recurrence. The importance of measuring the TT and DI in all the three coordinates (X, Y and Z) is highlighted.

## Material and Methods

- Case selection

Formalin fixed paraffin embedded tissue blocks of 14 confirmed cases of MISCC of tongue were retrieved from the departmental archives. The informed consent and approval from an ethics committee was obtained (IEC 407/2013). Clinical data obtained from the patients’ medical records revealed that 9 were males and 5 were females with a very wide age range from 20 to 78 years. Clinically these cases were staged T1/2N0M0 at the time of the initial diagnosis and histologic ally signed out as MISCC following biopsy. All the cases included in the study confirmed the Barnes’ criteria of MISCC (3). Treatment included conservative surgical excision with 0.5cm of margin clearance. Follow up of these cases for 5 or more years after surgery revealed that 10 patients remained disease free while 4 developed local recurrence.

- Methodology 

**Figure 1:**
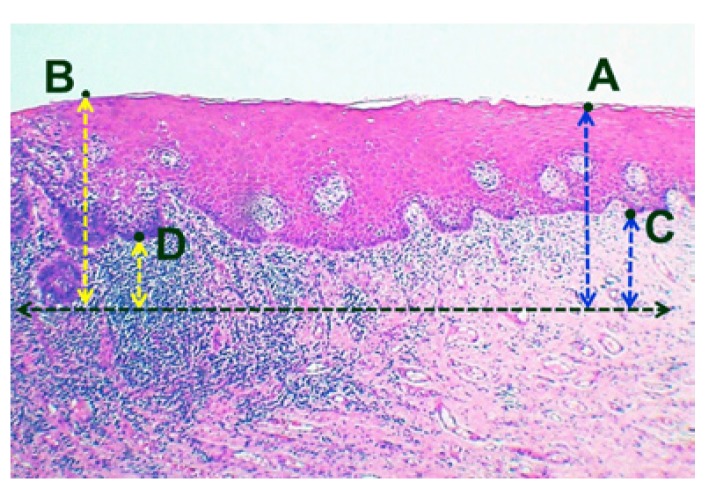
Representative image showing reference points for the measurement of TT (A, B) and DI (C, D) in MISCC (×25).

The haematoxylin and eosin (H and E) stained tissue sections of all the 14 cases were observed under light microscope with a 2.5x objective. The TT and the DI were measured from four distinct reference points (A-D). The first reference point was from the surface of the adjacent non-neoplastic epithelium (A) (5), the second was from the surface of histological invasion (B) (6), the third was from the basement membrane of the adjacent non-neoplastic epithelium (C) (7) and the fourth was from the basement membrane at the point of tumor infiltration (8) (D) (Fig. 1). TT and DI were calculated using an ocular micrometer (9) as well as image analysis software (Image Pro Insight). For ocular micrometry, the scale on ocular micrometer was standardized with the stage micrometer, with each division of ocular micrometer equaling 10µm of stage micrometer. All these measurements were recorded in micrometers (µm). While taking the measurements, the inflammatory component around the deepest point of invasion, as well as the keratinization of the surface epithelium, were excluded. These measurements were carried out two-dimensionally in the X and Y coordinates. The measurement of DI was carried out in the 3rd dimension for 4 cases that showed local recurrence as well as in 10 cases that did not recur. For this, the tissue blocks of cases that recurred were cut using a sharp instrument at 900 to the original plane/axis and were reoriented to obtain the Z axis. The true depth of invasion was ascertained using a computer aided 3D application (CATIA V5R19, version 5 release 19).

**Figure 2:**
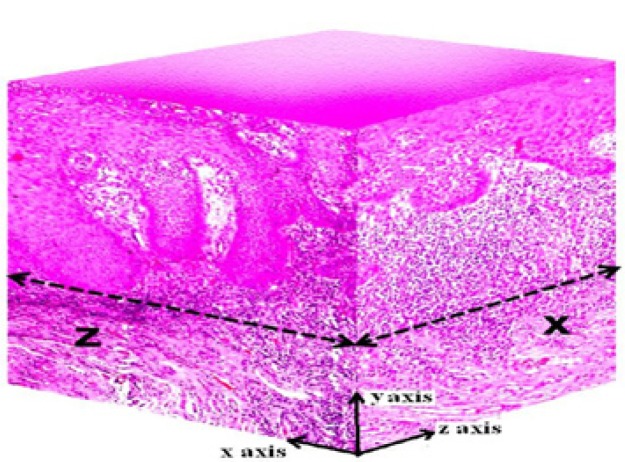
Recreation of XYZ coordinates using image editing software for 3D construction (×25).

**Figure 3:**
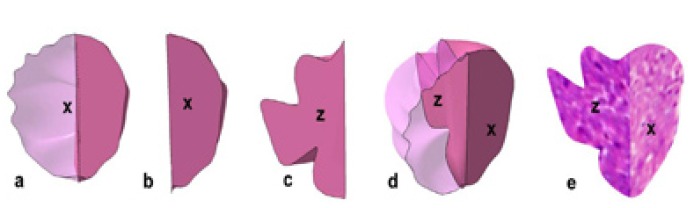
3D reconstruction of the tumor island shows a: 2D profile of the tumor island in XY axis; b: The cut up of one half the tumor island sectioned at 900; c: 2D profile of tumor island in Z axis; d: 3D profile of the tumor island on the X face with its complimentary part. e: Recreated 3D image of the tumor island depicting its true depth by imaging software.

The 3D reconstruction of the lesion was done on a Cartesian coordinate system and the measurement of DI in Z axis was carried out in the same way as in XY coordinate. All the measurements of the DI in X, Y and Z axis were recorded and compared. The images obtained were imported to modeling software to digitize the outline of the tumor. The coordinate data of the digitized outline were extracted to create a bounding curve. The true depth of tumor invasion could be ascertained using a computer aided 3D application utilizing the readings in two different coordinates. X face, as depicted in figure. 2, was on the YZ plane of the coordinate system and Z face was on the XY plane and this offered a better visualization of difference with regard to the depth of invasion in two different axes. The 3D reconstruction of tumor island from a case of MISCC that recurred was done using this software application (Fig. 3). The 2D profile of the tumor island in XY axis is shown in figure. 3a. One half of the specimen after sectioning at 90° is shown in figure. 3b. The 2D profile of tumor in Z axis is shown in figure 3c. The 3D profile of the tumor island on the X face with its complementary part is shown in figure. 3d. The reconstruction of the complimentary part of pathology on the Z face is not shown so as to provide the internal details of the same. The recreated image of the actual tumor island with its true depth in Z axis is shown in figure. 3e.

Mann-Whitney test was used to compare median TT and DI with gender, tumor stage and recurrence of the tumor. Wilcoxon Signed Ranks test was used to compare the median DI measurements of XY and Z coordinates measured by both the manual and the automated method as well as to compare the median measurements of XY and Z coordinate in cases that recurred and those that did not.

## Results

The measurements of TT and DI in XY coordinate for all the 14 cases including the measurement of TT and DI for 4 cases that recurred are presented in  Table 1. It was observed that the measurements with ocular micrometry and image analysis software did not show a significant variation. The association of TT and DI with gender, stage of tumor and recurrence was statistically insignificant (Mann-Whitney U test, *p*>0.05). The measurement of the DI by ocular micrometry and image analysis software in all the 14 cases in both the XY as well as in the Z coordinate are shown in  Table 2. Significantly, the DI in all the 4 cases that recurred had a greater DI in the Z-axis than in the XY-axis (values highlighted). The measurement of DI assessed in the Z coordinate for 10 cases that did not recur was lower than that in the XY axis. Wilcoxon Signed Ranks test revealed a statistically significant difference between the median DI measurements of XY and Z coordinates using the manual method (*p* = 0.048), while the measurements of the DI by automatic method showed no statistically significant difference in the median DI (*p* = 0.084) ( Table 3).

**Table 1:**
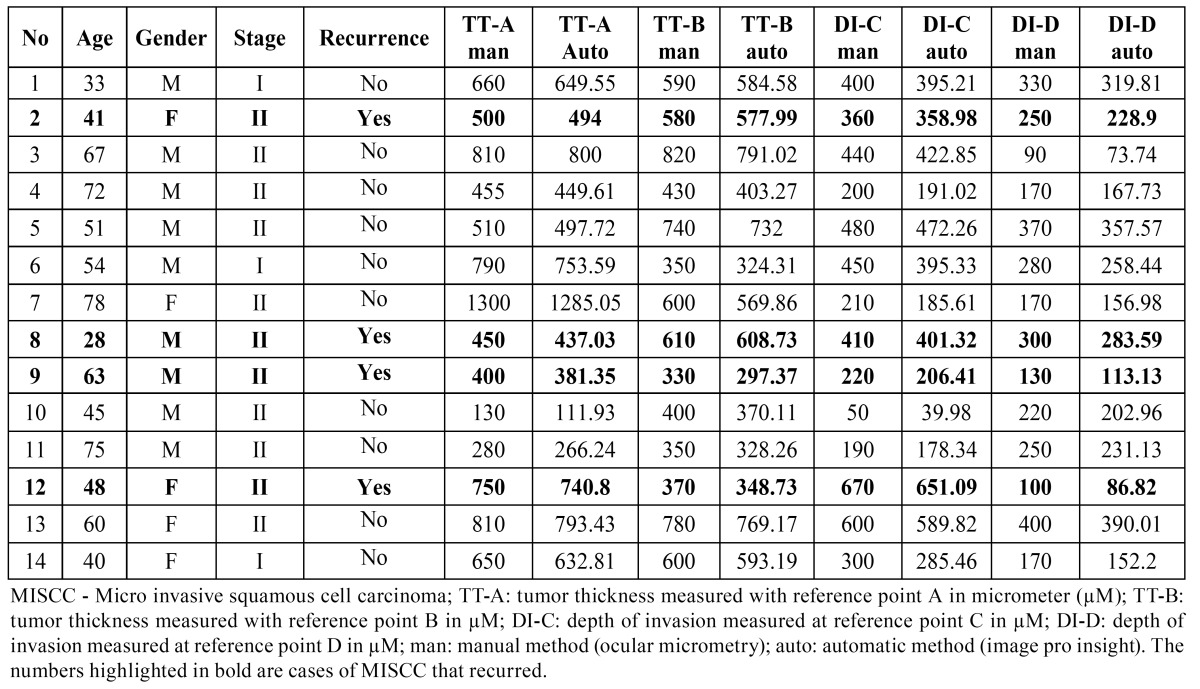
The patient demographics and the measurements of TT and DI in cases of MISCC by using both manual and automatic methods in XY co-ordinate.

**Table 2:**
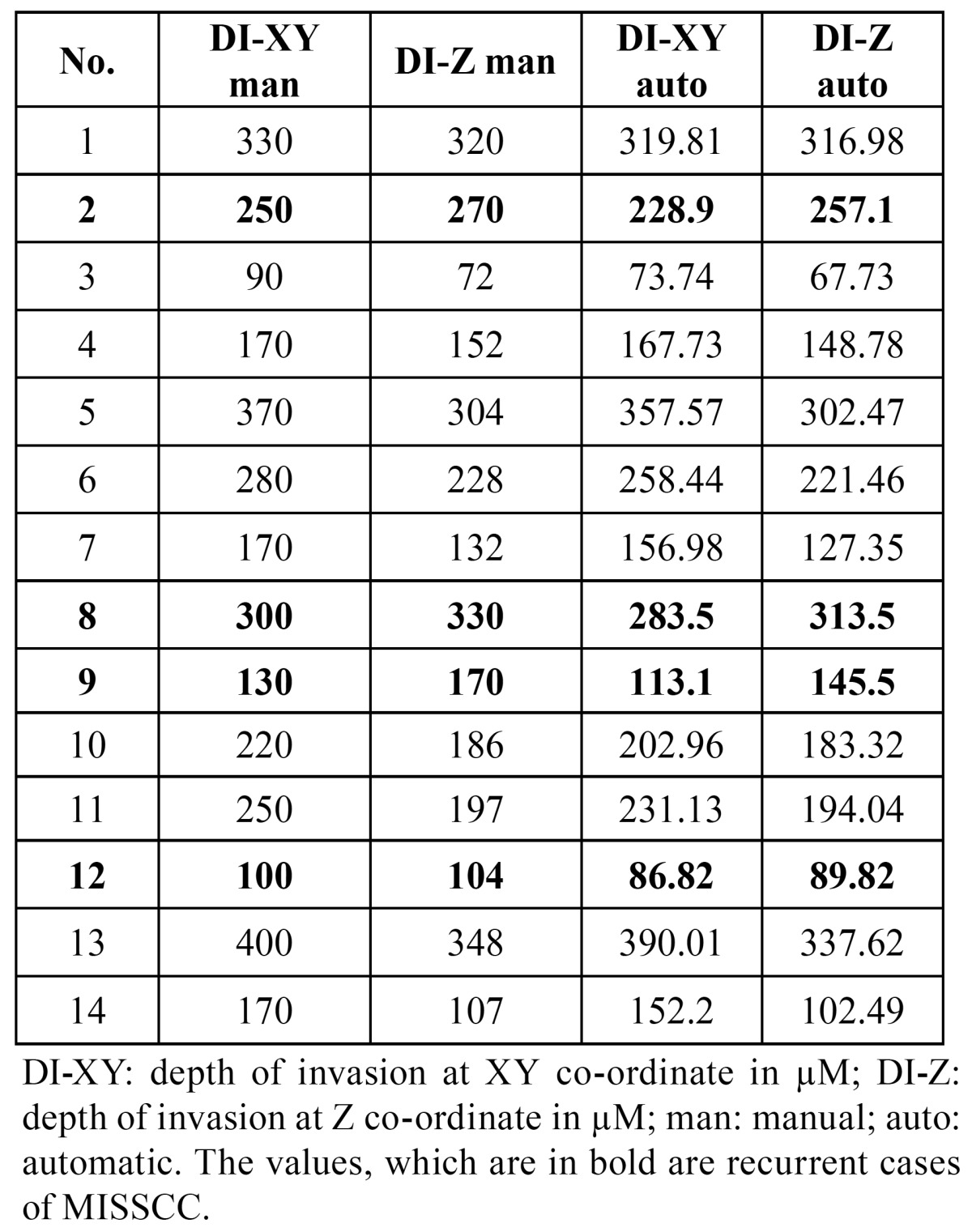
The measurement of the DI at reference point D in both the XY and Z axis in all cases.

**Table 3:**
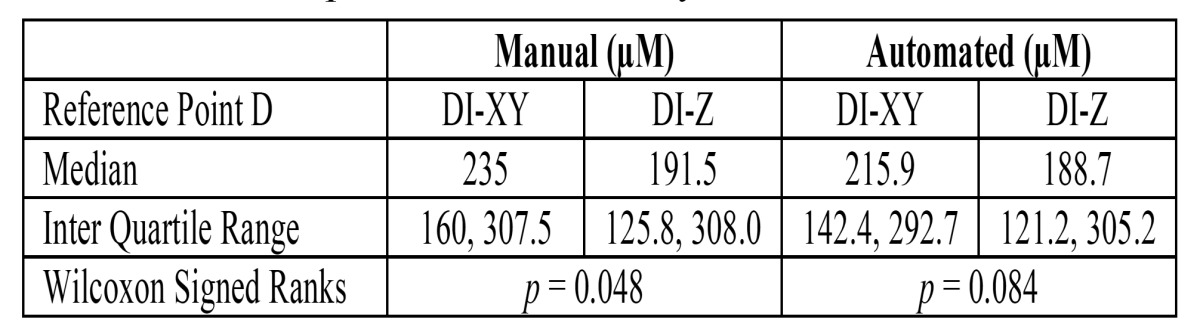
Comparison of the median DI measurements of XY and Z coordinates at point D measured by two methods.

## Discussion

Tongue is the most common intraoral site for malignancy, as per comparative worldwide studies (10-12) and tongue cancers have an increased risk of metastasis and recurrence (13). The depth of invasion along with the tumor volume, are frequently reported to be associated with increased risk of local recurrence. DI and TT are thus imperative in predicting the recurrence and survival in cases of TSCC (14). Loss of the basement membrane is the sine qua non of an invasive neoplasm. Adhesion, proteolysis and migration facilitate this process of invasion (15). Isolated cells, small circumscribed groups of cells, or irregularly shaped finger-like processes are some of the earliest histologic feature of invasion.

Micro invasive cancer is defined by the American Joint Committee on Cancer (AJCC) and Union for International Cancer Control (UICC) as a lesion that is predominantly intraepithelial with a focus of invasion (2) of microscopic dimensions confined to the superficial stroma or lamina propria. Barnes defines micro invasive carcinoma as invasive squamous cell carcinoma that extends into the stroma by <0.5 mm, as measured from the adjacent (non-neoplastic) epithelial basement membrane (3). The cases, in the current study, were selected as per Barnes’ criteria. However, the referral points with regard to MISCC could vary depending on the site of involvement. The depth of less than 5mm in cervical carcinomas (16) and 1mm in breast carcinoma, are considered as being micro invasive.

Clarity regarding the concepts of DI and TT in TSCC is important as these measurements have prognostic implications. Incidentally, these two terms have been used synonymously (8). While the DI is the extent to which the tumor cells are microscopically discernible beneath the epithelium, TT concerns the entire tumor volume. The tumor having the ability to expand facilitates the spread to proximal blood vessels and lymphatics, thereby increasing the risk of nodal metastases. It is therefore advisable to take the actual mass present beneath the theoretical reconstruction of a basement membrane (DI) rather than the thickness of tumor (TT) (8).

In the OSCC, the DI has been evaluated with an optical micrometer utilizing several approaches, irrespective of whether the mucosal surface, the tumor surface, or the ulcer base are chosen as the starting point (17). Breslow, while defining the criteria for measuring the depth of invasion in cutaneous melanoma, included the deepest point of invasion to the top of the granular cell layer of overlying epidermis, excluding keratin, parakeratin, and inflammatory exudates (6). If the lesion was ulcerated, the ulcer base would serve as the reference points.

Two factors which are of utmost importance for assessing the depth of tumor invasion are judicious sampling and appropriate sectioning. Another pertinent aspect with regard to precise categorization of the DI in MISCC is the cutoff measurement. This measurement is quite arbitrary and can range from 1.5mm to 10mm (18-20). One possible explanation for this discrepancy lies in the disparate techniques adopted by the pathologists to measure the DI. However, there is no clarification on the cut off measurement, which is a critical parameter on the disease outcome.

For many surgeons an adequate margin during treatment planning is 0.5cm. However, the decision to determine the most accurate margin is confounded by several factors. Significantly, the post fixation shrinkage as it constitutes for 30% of the tumor volume (21) and margin shrinkage of 3.7mm in tongue cancer ascribed to sudden contraction of the underlying musculature (22). Additionally the amount of margin shrinkage is higher in T1 and T2 tumors. More so, there is no clarity on margins exhibiting dysplasia. Taking all these factors into consideration, an average of 8-10mm of in situ margins is indispensable to obtain 5mm pathologically clear tumor margins.

Disruption of the basement membrane is generally agreed to be the prime criteria for the diagnosis of micro invasion. At a ultramicroscopic level, the basement membrane is usually absent, or if present is in a defective form in invasive cancer (23). Small cytoplasmic protrusions of tumor cells into the adjacent stroma can be seen in sites where the membrane is absent, seemingly representing cancer invasion. These findings were confirmed by Ashworth *et al*. (24) who also stated that invasive cell groups are partially or completely invested by a basement membrane that appears to be newly formed by cells of the invading peg.

The invasive peg in MISCC is characterized by a richness in cytoplasm and nuclei that are larger and clearer than those of the intraepithelial portion of the lesion. Some of the cells may be in various stages of degeneration in association with leucocytic infiltration. Small tongues of tapering or branching epithelial cells, as well as the formation of narrow cell columns or the appearance of cell groups that seem to be invading the stroma were also considered as criteria for invasion (25).

The most reliable measurement in our study was the one measured from the basement membrane of the tumor to the deepest point of invasion (D). However, the DI varies with the level of sectioning in the two dimensional measurement. To overcome this limitation, step sectioning with cone biopsy has been preferred in cases of micro invasive carcinoma of cervix, which involves preparation of minimum of 15 blocks from the given biopsy tissue and removal of eight to ten sections at intervals through each block (26). Alternatively, dismantling the cone into two wedge-shaped blocks and preparing two sections from each block has also been considered. If any of the two sections revealed frankly invasive carcinoma, no further investigation was necessary, but if micro invasion was observed then the block would be further step sectioned, resulting in a total of 100-150 sections (27). However, this proposed methodology of measurement of DI has never obtained wide acceptance as it requires examination of numerous serial sections.

Due to this inadequacy of two dimensional measurement, we ascertained the DI using the third coordinate i.e. Z-axis and reconstructed the 3D image of tumor island in question using a specially designed 3D reconstruction software, taking into consideration, the length, breadth and depth of the lesion. Following the assessment of the lesion and marking its depth of invasion in the X plane, the Z plane was created by sectioning the block through the center of the lesion in question to evaluate its true depth from the deepest part in the X plane. The creation of Z plane was to assess at least in one plane the true depth of invasion.

The 4 cases that recurred showed a greater DI in Z coordinate compared to the X coordinate. The assessment of DI in the X axis was inappropriate as the DI in Z coordinate was greater and an inadequate removal of tumor thereafter could have led to their recurrence. As it was a case series of 14 patients, actual cutoff point for MISCC could not be established and also the area under the receiver operating characteristics (ROC) could not be defined. The computer aided three-dimensional reconstruction of the tumor in a series of cases of MISCC includes the tumor as a whole than as part.

## Conclusion

Precise knowledge of the DI allows complete removal of tumor and thus improves prognosis. The assessment of the depth of tumor in Z axis is critical for accurate assessment of MISCC of tongue. However, this alone does not qualify as the most reliable method of tumor prognosis. The development of efficient software to visualize the 3D reconstruction of histological images in many slices, along with the assessment of the tumor micro environment, may add validity to these methods.
